# Relocation of inadequate resection margins in the wound bed during oral cavity oncological surgery: A feasibility study

**DOI:** 10.1002/hed.25690

**Published:** 2019-02-01

**Authors:** Cornelia G.F. van Lanschot, Hetty Mast, Jose A. Hardillo, Dominiek Monserez, Ivo ten Hove, Elisa M. Barroso, Froukje L.J. Cals, Roeland W.H. Smits, Martine F. van der Kamp, Cees A. Meeuwis, Aniel Sewnaik, Rob Verdijk, Geert J.L.H. van Leenders, Vincent Noordhoek Hegt, Tom C. Bakker Schut, Robert J. Baatenburg de Jong, Gerwin J. Puppels, Senada Koljenović

**Affiliations:** ^1^ Department of Otorhinolaryngology and Head and Neck Surgery, Erasmus MC University Medical Center Rotterdam Rotterdam The Netherlands; ^2^ Center for Optical Diagnostics and Therapy, Department of Dermatology, Erasmus MC University Medical Center Rotterdam Rotterdam The Netherlands; ^3^ Department of Oral and Maxillofacial surgery, Erasmus MC University Medical Center Rotterdam Rotterdam The Netherlands; ^4^ Department of Pathology, Erasmus MC University Medical Center Rotterdam Rotterdam The Netherlands

**Keywords:** intraoperative assessment, oral cavity, relocation, resection margin, specimen driven

## Abstract

**Background:**

Specimen‐driven intraoperative assessment of the resection margins provides immediate feedback if an additional excision is needed. However, relocation of an inadequate margin in the wound bed has shown to be difficult. The objective of this study is to assess a reliable method for accurate relocation of inadequate tumor resection margins in the wound bed after intraoperative assessment of the specimen.

**Methods:**

During oral cavity cancer surgery, the surgeon placed numbered tags on both sides of the resection line in a pair‐wise manner. After resection, one tag of each pair remained on the specimen and the other tag in the wound bed. Upon detection of an inadequate margin in the specimen, the tags were used to relocate this margin in the wound bed.

**Results:**

The method was applied during 80 resections for oral cavity cancer. In 31 resections an inadequate margin was detected, and based on the paired tagging an accurate additional resection was achieved.

**Conclusion:**

Paired tagging facilitates a reliable relocation of inadequate margins, enabling an accurate additional resection during the initial surgery.

## 1 INTRODUCTION

Surgery is one of the main treatment modalities for oral cavity cancer. The goal is complete tumor removal with adequate resection margins (i.e. more than 5 mm of healthy tissue between tumor border and resection surface).^1^ At the same time, healthy tissue should be spared as much as possible to preserve function and esthetics.

Of all oncological prognostic factors (i.e. patient and tumor characteristics), physicians can only influence the quality of resection margins. Inadequate resection margins negatively influence local recurrence, the need for adjuvant therapy, and patient survival.^2^, ^3^, ^4^ Even the presence of dysplasia of squamous epithelium in the resection margins is associated with a higher risk on local tumor recurrence.^1^, ^5^, ^6^ For that reason, at our institute the resection margins containing severe dysplasia/in situ carcinoma is considered inadequate as well.

In the oral cavity, an adequate tumor resection is often hard to achieve because of the complex anatomy, the demand for satisfactory remaining function, and acceptable physical appearance. During tumor resection, the surgeon relies only on his/her eyes and hands, and preoperative imaging. For oral cavity squamous cell carcinoma (OCSCC) surgery, recent studies show poor results with an adequate tumor resection in only 15% of the cases.^2^, ^3^ Evidently, inspection and palpation are not sufficient to distinguish between tumor and the surrounding healthy tissue. In order to control resection margins, intraoperative assessment based on the frozen section procedure is available. Of all surgical disciplines, intraoperative assessment of the resection margins is most often used in head and neck surgery.^7^ Except for Mohs surgery, the role of the frozen section procedure in other surgical fields is limited. During intraoperative assessment of resection margins by frozen section analysis, suspicious tissue is usually sampled from the wound bed by the surgeon, therefore the method is also called wound bed/defect‐driven assessment. In recent years, the specimen‐driven assessment, in which the surgeon and pathologist together assess the resection margins on the specimen, has been advocated. There is growing evidence that a specimen‐driven assessment is superior to wound bed‐driven assessment due to better visualization and less sampling error^4^, ^8^, ^9^, ^10^ (Smits et al, unpublished data, 2018). Based on this evidence, the American Joint Committee on Cancer (AJCC) adopted specimen‐driven intraoperative assessment as standard of care in the current guidelines.^11^

Although intraoperative assessment can be beneficial with both specimen‐driven and wound bed‐driven, either method lacks an accurate relocation of the inadequate margin. It is known that relocation is particularly difficult in the head and neck region, and therefore an optimal additional resection is not always achieved.^9^, ^12^, ^13^, ^14^, ^15^, ^16^, ^17^, ^18^, ^19^

Various ideas to solve the problem of relocation of inadequate resection margins have been described, but none of them seems to be efficient. For the wound bed‐driven assessment, the use of surgical clips in the wound bed is frequently reported,^20^, ^21^, ^22^ as well as systematic cavity shavings, in which tissue is sampled for frozen sections by shaving the wall of the surgical cavity.^23^, ^24^ For specimen‐driven assessment, Mohs' surgery^25^, ^26^ or mapping of the margins (e.g. Breuninger technique)^27^, ^28^ are successfully used in dermato‐oncology, which also harbors the problem of relocation. Although it has been described recently for small and simple OCSCC resection specimens, this method is not applicable for all head and neck resection specimens.^29^

The main goal of the current study was to report on a reliable and objective method for relocation of the inadequate margins from specimen to the wound bed, based on intraoperative specimen‐driven assessment, and to assess the ease and accuracy of this method in the surgico‐pathological workflow.

## 2 MATERIALS AND METHODS

**Figure 1:**
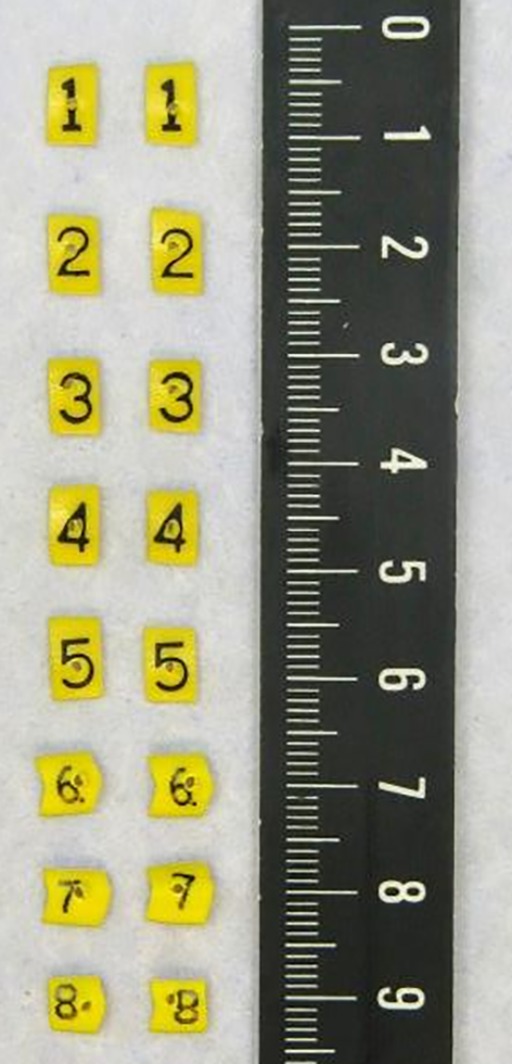
Tags [Color figure can be viewed at wileyonlinelibrary.com]

**Figure 2:**
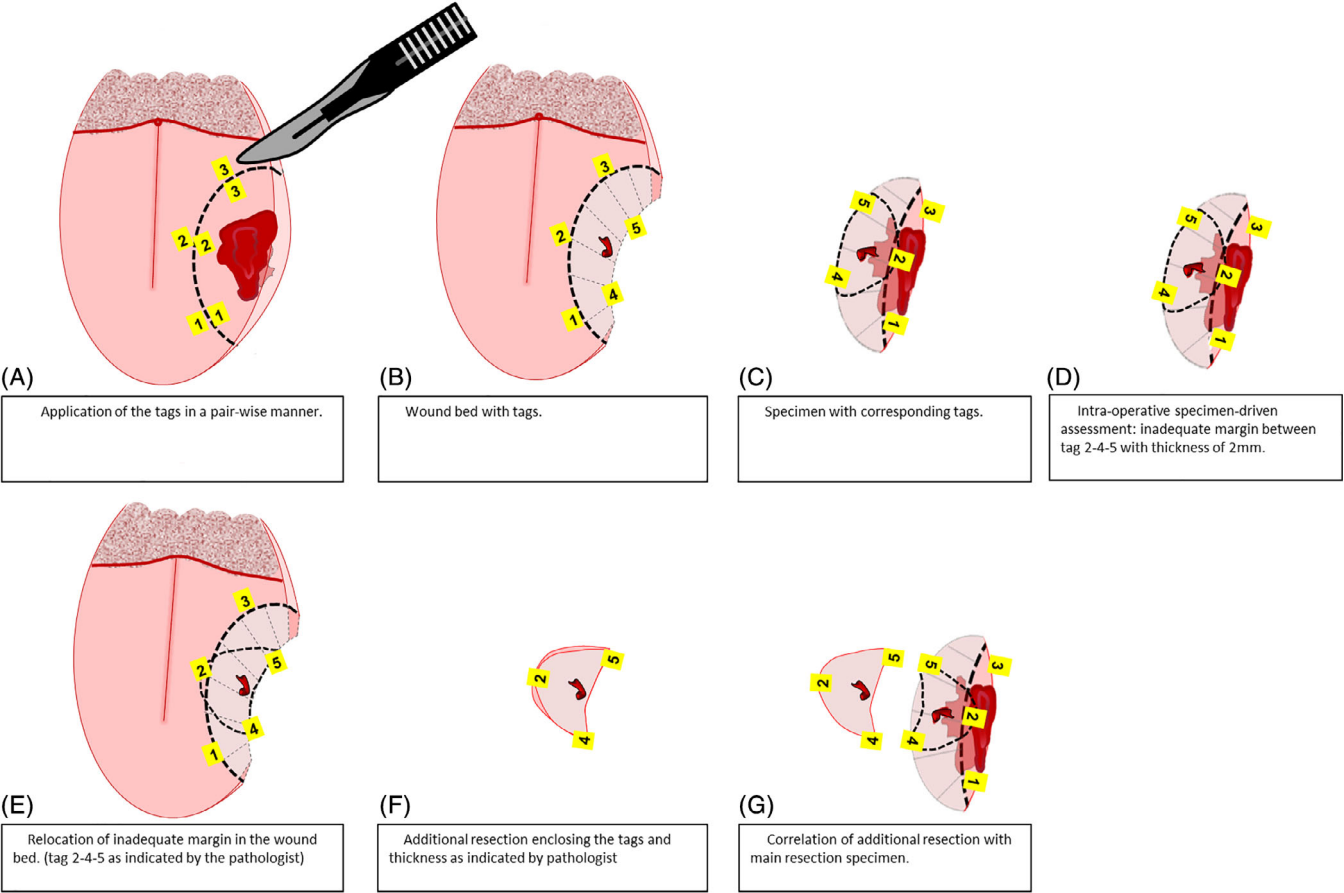
Paired tagging method, overview. A, Application of the tags in a pair‐wise manner. B, Wound bed with tags. C, Specimen with corresponding tags. D, Intraoperative specimen‐driven assessment: inadequate margins between tag 2‐4‐5 with thickness of 2 mm. E, Relocation of inadequate margins in the wound bed. (Tag 2‐4‐5 as indicated by the pathologist.) F, Additional resection enclosing the tags and thickness as indicated by pathologist. G, Correlation of additional resection with main resection specimen

The study was approved by the Medical Ethics Committee of the Erasmus MC Cancer Institute, Rotterdam, the Netherlands (MEC‐2017‐1016). In recent years, at the Erasmus MC Cancer Institute, we use a paired tagging method for relocation of the inadequate margins from the specimen to the wound bed in oral cavity cancer surgery. Patients with a primary or recurrent tumor of the oral cavity were included for this method. The tags (Premier Farnell Limited BV, Utrecht, the Netherlands), numbered from 0 to 9, were cut to a size of 5 mm × 7 mm × 2 mm. The tags were perforated in order to fix the tag with a suture into the tissue (Figure 1). The tags were sterilized in alcohol 60 minutes before the surgery. During resection, the surgeon fixed the tags with the same number in a pair‐wise manner, along both superficial and deep resection lines. In this way, one tag of each pair remained on the resection specimen and the other tag in the wound bed. The tagging procedure is illustrated in Figure 2A‐C.

**Figure 3:**
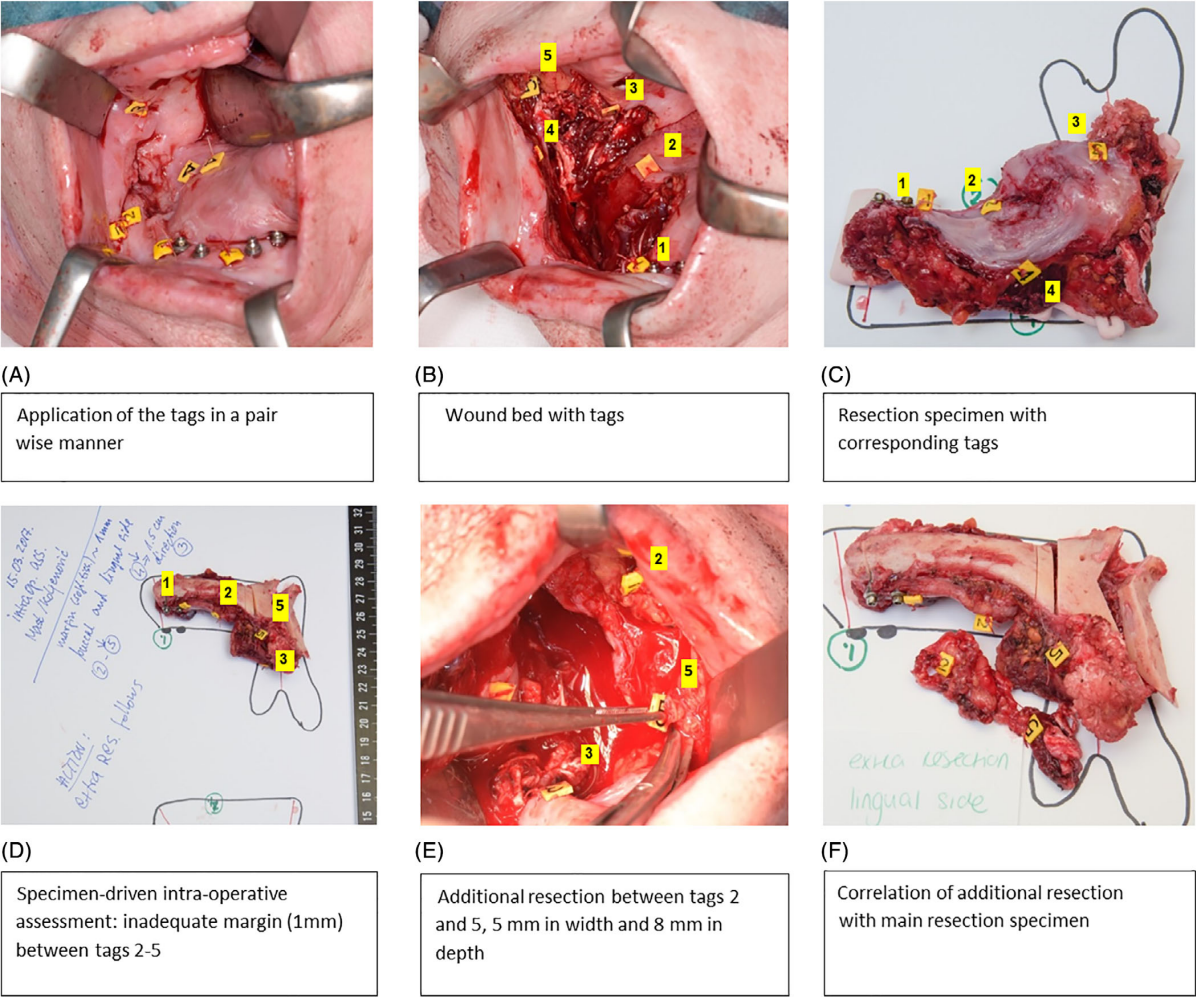
Paired tagging, including intraoperative assessment of the resection specimen and correlation of the additional resection with the resection specimen. A, Resection of the tumor of the right processus alveolaris with application of the tags in a pair‐wise manner. B, Wound bed with numbered tags (superficial and deep). C, Resection specimen with corresponding numbered tags. D, Intraoperative specimen‐driven assessment of the resection margins; an inadequate margin was found between tags 2‐5. E, An additional resection based on relocation, enclosing the corresponding tags and thickness, as indicated by the pathologist. F, Assessment of the accuracy of the additional resection based on correlation based on with main specimen

A specimen‐driven intraoperative assessment of the resection margins was followed as standard procedure. The pathologist and the surgeon together assessed the resection specimen by inspection (visually and by palpation) and by incisions perpendicular to the resection plane. If the tumor border could not be clearly identified by visual inspection, the assessment was refined by frozen section histopathology. The resection margins for invasive tumor are defined as adequate; more than 5 mm of healthy tissue between tumor border and resection surface, or inadequate; less than 5 mm of healthy tissue between tumor border and resection surface, in accordance with the guidelines of the Royal College of Pathologists. Moreover, according to our institutional guidelines, resection margins containing severe dysplasia/in situ carcinoma are also classified as inadequate. In all cases in which margins were adequate, the tags were removed from the wound bed. If an inadequate margin was found, the numbered tags enclosing this area on the resection specimen indicated its location. Moreover, desirable thickness/depth of the additional resection, to achieve an adequate margin, was also indicated by the pathologist (in millimeters), depending on the initial margin. For example, if initial margin was 2 mm, the pathologist recommended an additional resection of tissue with at least 5 mm thickness. Based on this information, the surgeon relocated the corresponding tags in the wound bed and performed an additional resection around these tags with the indicated tissue thickness. The accuracy of the relocation method was checked by comparing the numbers of the tags on the additional resection specimen with the numbers of the tags surrounding the inadequate margin on the main specimen. No intraoperative assessment of the margins in the additional resection was performed. An illustration of the relocation method from the specimen to the wound bed is shown in Figure 2D‐G. In Figure 3, an example of the tagging method with additional resection, during an “en bloc” resection with segmental mandibular resection, based on relocation with paired tags is shown, including the correlation of the additional resection with the main resection specimen. After correlation of the additional resection with the main resection specimen, the remaining tags were removed from the wound bed. After completion of surgery, the main specimen and any additional resection specimen followed the standard pathological procedure. Information regarding specimen characteristics, type of surgery, and status of resection margins based on intraoperative assessment were collected. The number of tags used and their exact location were recorded during each surgery. The time needed for placing the tags was also recorded. In addition, the ease of placing the tags and the ease of relocation of inadequate margins in the surgical wound bed were documented. The ease and accuracy of the correlation of the additional resection with the main specimen were also recorded.

## 3 RESULTS

From September 2015 until September 2017, the method of paired tagging, as described in the previous section, was applied during 80 surgeries (79 patients) for oral cavity tumors, at the Erasmus MC Cancer Institute. The group comprised 78 squamous cell carcinomas and 2 salivary carcinomas (1 mucoepidermoid carcinoma and 1 adenoid cystic carcinoma). Most of the tumors were early stage carcinomas (20 cT1 and 29 cT2). From 80 surgeries, there were 30 (37%) local resections, 15 (19%) “en bloc” resections, 16 (20%) “en bloc” resections with segmental mandibular resections, 8 (10%) “en bloc” resections with marginal mandibular resections, 7 (9%) hemiglossectomies, 2 (2.5%) subtotal glossectomies, and 2 (2.5%) were partial maxillectomies. In all cases, specimen‐driven intraoperative assessment of the resection margins was performed. None of the patients had received radiation therapy prior to surgery.

A maximum distance of 5 mm between the two tags of one pair was maintained. For local excisions, four to five tag pairs were sufficient, with an interval of 1 cm between different tag pairs. In case of large resections, usually all 10 tag pairs were used (numbered 0‐9) which were fixed with intervals of approximately 2‐3 cm between different tag pairs. The time needed to suture one tag was on average 30 seconds. The surgeons reported an easy relocation of the inadequate resection margin from specimen to the wound bed. They described the use of the tags as easy but time consuming, and therefore interfering with the surgical workflow (H. Mast, MD, DDS; J. A. Hardillo, PhD; D. Monserez, MD; I. ten Hove, MD, DDS; C. A. Meeuwis, PhD; A. Sewnaik, PhD; R. J. Baatenburg de Jong, PhD, oral communication, September 2015‐September 2017). The pathologists reported that the tags enabled accurate anatomical orientation of the specimen. Moreover, pairing of the tags on the resection specimen and the additionally resected tissue enabled the pathologists to determine that an as accurate as possible additional resection has been performed. In general, the pathologists did not experience any obstruction of the pathological workflow by this method (R. Verdijk, PhD; G.J.L.H. van Leenders, PhD; S. Koljenović, PhD, oral communication, September 2015‐September 2017). Both the surgeons and pathologists described the method, also referred as Erasmus MC relocation method, as indispensable. Currently, the method has been used as standard of care during head and neck surgery at our institution. Moreover, there is a great interest in this relocation technique by other centers, nationally and internationally.

**Figure 4:**
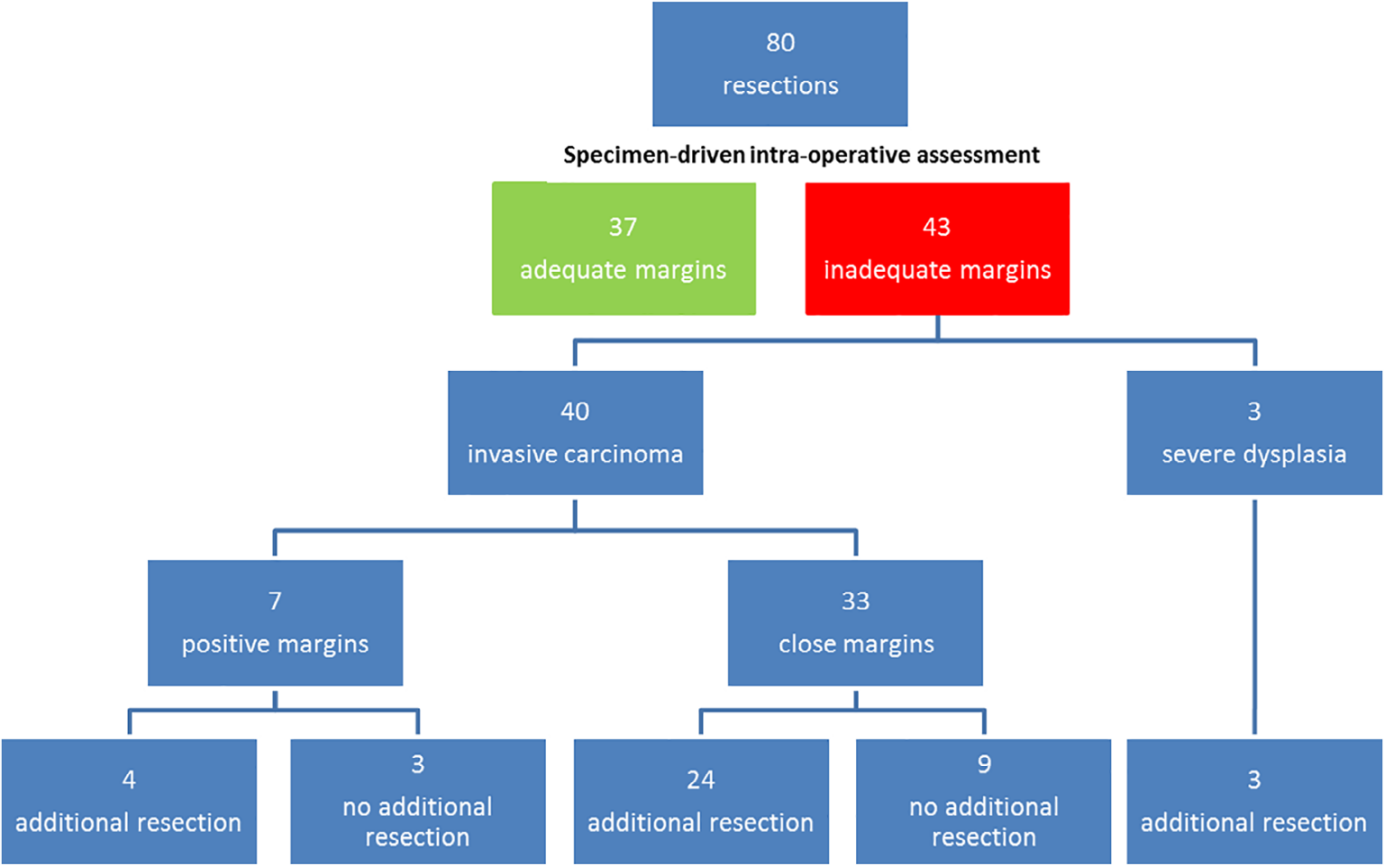
Overview surgico‐pathological workflow based on specimen‐driven intraoperative assessment of resection margins

During intraoperative specimen‐driven assessment, in 43 of 80 cases an inadequate margin was found for invasive carcinoma (7 tumor‐positive margins, 33 close margins) and for severe dysplasia (3 cases with dysplasia‐positive mucosal margins). In 31 of these cases, an additional resection was performed based on the relocation method: 4 for tumor‐positive margins, 24 for close margins, and 3 for severe dysplasia. In the remaining 12 cases (3 tumor‐positive margins, 9 close margins), additional resection was not performed for different reasons: in 11 cases because additional resection interfered with maintenance of function and esthetics (e.g. overlying skin or mandible), and in 1 case it was not possible due to the close relation with the internal carotid artery. The results are summarized in Figure 4.

After additional resection, final pathology confirmed that in 28 out of the 31 cases, the status of that specific resection margin was improved: in 25 cases an adequate margin was obtained, and in 3 cases the revised margins were improved from 0.1 to 2.1 mm, from 1 to 4.7 mm, and from 2 to 3 mm. In the last three cases, the margins remained tumor positive. These data are shown in Table 1.

**Table 1:** Characteristics of resection specimen with revised margins based on Erasmus MC relocation method

Case number	Location tumor	Type of surgery	Intraoperative assessment: resection margins	Additional resection margin	Accurate additional resection achieved
1	Tongue	Local excision	Dysplasia (3 mm)	Clear (7 mm)	Yes
2	Buccal mucosa	“En bloc” resection with segmental mandibular resection	Dysplasia (4 mm)	Clear (6 mm)	Yes
3	Floor of the mouth	“En bloc” resection with marginal mandibular resection	Dysplasia (4 mm)	Clear (8 mm)	Yes
4	Tongue	Local excision	Close (<5 mm)	Clear (7 mm)	Yes
5	Floor of the mouth	“En bloc” resection	Close (2 mm)	Close (3 mm)	Yes
6	Oropharynx	Local excision	Close (1.5 mm)	Clear (5.5 mm)	Yes
7	Tongue	Subtotal glossectomy	Close (1.8 mm)	Clear (5.8 mm)	Yes
8	Floor of the mouth	Local excision	Close (1 mm)	Close (4.7 mm)	Yes
9	Mandible	“En bloc” resection with segmental mandibular resection	Close (1 mm)	Clear (5.5 mm)	Yes
10	Floor of the mouth	“En bloc” resection	Close (1 mm)	Clear (5.5 mm)	Yes
11	Tongue	Local excision	Close (1 mm)	Clear (6 mm)	Yes
12	Alveolar process	“En bloc” resection with segmental mandibular resection	Close (2 mm)	Positive (<0.1 mm)	Yes
13	Tongue	Local excision	Close (2 mm)	Clear (6 mm)	Yes
14	Tongue	Local excision	Close (2 mm)	Clear (6 mm)	Yes
15	Floor of the mouth	“En bloc” resection with marginal mandibular resection	Close (2 mm)	Clear (7 mm)	Yes
16	Trigonum retromolare	“En bloc” resection with segmental mandibular resection	Close (2 mm)	Clear (7 mm)	Yes
17	Alveolar process	“En bloc” resection with segmental mandibular resection	Close (3.1 mm)	Clear (5.1 mm)	Yes
18	Tongue	“En bloc” resection	Close (3.5 mm)	Clear (9 mm)	Yes
19	Alveolar process	“En bloc” resection with segmental mandibular resection	Close (3 mm)	Clear (13 mm)	Yes
20	Floor of the mouth	“En bloc” resection with marginal mandibular resection	Close (3 mm)	Clear (6 mm)	Yes
21	Alveolar process	“En bloc” resection with segmental mandibular resection	Close (3 mm)	Clear (8 mm)	Yes
22	Buccal mucosa	Local excision	Close (4 mm)	Positive (0.1 mm)	Yes
23	Tongue	Local excision	Close (4 mm)	Clear (11 mm)	Yes
24	Tongue	“En bloc” resection	Close (4 mm)	Clear (6 mm)	Yes
25	Floor of the mouth	“En bloc” resection	Close (4 mm)	Clear (8 mm)	Yes
26	Tongue	Hemiglossectomy	Close (4 mm)	Clear (8 mm)	Yes
27	Tongue	Local excision	Close (4 mm)	Clear (9 mm)	Yes
28	Buccal mucosa	Local excision	Positive (<0.1 mm)	Close (2.1 mm)	Yes
29	Base of the tongue	“En bloc” resection	Positive (<1 mm)	Positive (<0.1 mm)	Yes
30	Tongue	Hemiglossectomy (“en bloc”)	Positive (<1 mm)	Clear (5.6 mm)	Yes
31	Alveolar process	Partial maxillectomy	Positive (<1 mm)	Clear (13 mm)	Yes

Two patients with a second resection because of recurrent disease were included in this study. In both cases, the initial margin was inadequate and was improved to adequate after additional resection. Post operative radiotherapy (PORT) was given based on the following guidelines: with main criteria comprising positive resection margins, lymph node metastases with extra nodal extension, or ≥2 positive lymph nodes. Minor criteria are close resection margins, infiltrative growth, perineural growth, and pT3/T4. Twelve patients received PORT, based on the above‐mentioned guidelines. Two patients had an indication for PORT but refused the treatment.

## 4 DISCUSSION

Intraoperative assessment of the resection margins is only meaningful if an accurate additional resection is enabled.

McIntosh et al. described that intraoperative control of the resection margins is more frequently performed in head and neck surgery than in other surgical specialties.^7^ According to the current guidelines of the AJCC, specimen‐driven intraoperative assessment is the standard of care.^11^ Although powerful, the impact of intraoperative assessment is negatively influenced by the lack of accurate relocation of inadequate margins for optimal additional resection towards adequate surgery.^9^, ^12^, ^13^, ^14^, ^15^ As a result, various studies have reported an accurate additional excision for initial tumor‐positive margins in only 22.5%‐50% of the cases.^12^, ^16^, ^17^, ^18^ Kerawala and Ong performed a study on relocation of the site in the wound bed in which tissue was sampled for a frozen section procedure (during wound bed‐driven assessment). In this study, the surgeon was asked to indicate the sites of sampling. After 5 minutes, the same surgeon was asked to relocate each site. In 32% (23 of 71) there was an error of more than 1 cm. The authors concluded that, due to the complex anatomy of the head and neck region, and the three‐dimensional structure of the wound bed, it was difficult to relocate the exact place of the inadequate margin, especially in larger resections.^15^ Maxwell et al. found a disappointing high percentage of inadequate resection margins and low local recurrence‐free survival for patients with an additional resection based on specimen‐driven intraoperative assessment. These poor results were explained by the following author's statement: “owing to the challenges of relocating the exact aspect of the relevant margin in the tumor bed, size discrepancy, and uncertain orientation of the additional tissue, it is conceivable that, in some patients, the additional margin may not actually cover the entire residual tumor at the positive margin.”^9^ The importance of relocation was also highlighted by Williams et al.^13^ In this review, the impact of the additional resection was estimated by local control rates. Better local control (LR 13%‐18%) was found for the surgical resections with adequate margins on initial surgery (in which no additional resection was needed), compared to resections in which adequate margins were achieved after additional resection based on specimen‐driven intraoperative assessment (LR 22%‐32%). These authors also concluded that the imprecision of relocation might be a contributing factor to these increased local failures. They stated that another factor, complicating accurate relocation of inadequate margin in the wound bed, is the retraction of the muscle tissue which results in misrepresentation of original anatomical relationships.^13^ Also Hinni et al. reported that “defect disorientation” can limit an accurate relocation of inadequate margin.^10^

The method of paired tagging (with numbered tags) solves the various problems hampering the relocation as mentioned by many authors, such as (muscle) tissue retraction and wound bed deformation, leading to size discrepancy, and the complex anatomy of the three‐dimensional structures.^9^, ^10^, ^13^, ^15^ This relocation method with numbered tags is objective and enables clear communication between pathologist and surgeon. The results of this feasibility study presented here show that by paired tagging, an accurate additional resection was performed in all cases in which initial margin was inadequate. In one case, the initially tumor‐positive margin was revised to close margin. Although the additional resection may not always result in an adequate margin, it might have positive impact on the need for adjuvant treatment. A tumor‐positive margin is one of the main criteria for PORT, with or without chemotherapy. It is likely, therefore, that the additional resection, guided by the relocation method described here, will have the most impact for patients with pT1‐T2 tumors in which other minor criteria for adjuvant therapy are also absent (e.g. positive nodal status, extra nodal extension, perineural growth, and infiltrative growth). For two remaining cases, the margin remained close (1 ‐ 4.7 and 2 ‐ 3 mm). However, Nason et al. describe that each additional millimeter of tumor‐free margin may be beneficial for patient outcome.^30^ Although we present promising results of inadequate margin relocation, at this stage, the method has some limitations such as sterility for the use in all head and neck resections, duration of placing the tags, size of the tags, and interruption of the surgical workflow. In order to improve the procedure, we are now developing a prototype instrument for rapid and easy placement of the tags and for tag removal. The goal is to simplify implementation of the procedure, to make the tags 3 × 4 mm. Finally, we seek a tagging prototype and optimized protocol that can be used by surgeons in all other specialties. We are preparing a retrospective clinical cohort study with matched pair analysis consisting of a larger group of patients and sufficient follow‐up.

It can be concluded that this simple relocation method enables an accurate additional resection when an inadequate margin is found during intraoperative assessment. It is expected that the implementation of paired tagging will lead to a higher number of adequate tumor resection margins, and thereby will lead to a better patient outcome and/or reduce adjuvant therapy and the related morbidity.

## CONFLICTS OF INTEREST

None declared.
